# Successful Treatment of ANCA-Negative Wegener's Granulomatosis with Rituximab

**DOI:** 10.1155/2010/846063

**Published:** 2010-10-26

**Authors:** Afsha Khan, Catherine A. Lawson, Mark A. Quinn, Amanda H. Isdale, Michael J. Green

**Affiliations:** Department of Rheumatology, York Hospital, Wigginton Road, York YO31 8HE, UK

## Abstract

Wegener's Granulomatosis (WG) is a systemic vasculitis typically associated with antineutrophil cytoplasmic antibodies (ANCAs). A small proportion of patients are ANCA negative, however, and this is more commonly found in individuals with disease limited to the ears, nose, throat, and lungs, who do not have renal involvement. Rituximab is a monoclonal anti-CD20 antibody that has been demonstrated to be effective in the treatment of autoantibody-associated rheumatic diseases, including systemic WG. We report the case of a patient with ANCA-negative WG who responded well to rituximab, illustrating that even in the absence of detectable autoantibodies, B-cell depletion can be effective.

## 1. Introduction

Up to 40% of patients with WG whose disease is limited to ear, nose, throat (ENT), and lung manifestations are ANCA-negative [1]. The role of rituximab in the treatment of limited WG or ANCA-negative disease remains uncertain. We report a case of a 21-year-old female with ANCA-negative WG limited to ENT and lung involvement, who responded well to rituximab with resolution of symptoms, normalisation of inflammatory markers, and reduction of steroid therapy.

## 2. Case

A 20-year-old woman presented with a five-week history of otalgia, ear discharge, and deafness. There were no additional systemic symptoms or signs. Audiometry showed bilateral conductive hearing loss. She initially received antibiotics; however due to severe otalgia, she had bilateral myringotomies with grommet insertion without improvement. CT and MRI revealed fluid in the middle ear clefts and mastoid cells bilaterally. Blood tests showed a C-reactive protein (CRP) of 50 mg/l, normal full blood count, renal and liver function tests, and a negative ANCA. Urinalysis was negative. Oral prednisolone 60 mg daily resulted in some symptomatic improvement. Seven weeks later, she developed a left lower motor facial nerve palsy and reduced sensation in the middle third of her face. Repeat MRI showed bilateral middle ear inflammation but no additional intracranial abnormalities. Further oral steroids again improved her symptoms. Nasal and middle ear biopsies revealed evidence of a necrotising inflammatory process, but no features of infection.

Further oral prednisolone improved the clinical symptoms with some subjective improvement in the facial weakness and general well-being, and reduction in CRP. Reducing steroids to 20 mg resulted in deterioration of symptoms, with increasing facial and ear pain plus a rise in CRP. With this further deterioration despite corticosteroids, a decision was taken to escalate therapy and she received cyclophosphamide with methylprednisolone plus cotrimoxazole, with slow improvement noted in her left ear hearing, clinical symptoms, and CRP. After three pulses, a CT scan of her lungs was requested to investigate a persistent cough. Despite normal repeat chest radiographs, cavitating lung lesions consistent with a diagnosis of WG were demonstrated. Other possible differential diagnoses including infections such as tuberculosis or *Staphylococcus aureus* abscesses were considered less likely due to the clinical and biochemical improvement with immunosupppression. She also developed a saddle nose deformity at this time. Relapsing polychondritis was thought to be unlikely given the pulmonary manifestations and the absence of external ear cartilage involvement. Histology had not shown atypical lymphocytes or histiocytes and so was not suggestive of midline granulomatosis. 

Following six pulses of cyclophosphamide, she developed stridor, and a flow volume loop was in keeping with a subglottic tracheal stenosis. She required multiple tracheal dilatations plus local steroid injections which brought temporary relief for approximately six to eight weeks at a time. After a further three pulses of cyclophosphamide, she remained well with no symptoms of active disease and a normal CRP. However, seven weeks after finishing cyclophosphamide, she developed pain over the right mastoid and maxillary sinus, shortness of breath, recurrent tracheal stenosis, increasing saddle nose deformity, and a rise in CRP to 26 mg/l. Despite her ANCA negativity, we felt that rituximab was a reasonable therapeutic option. She received two 1 g doses of rituximab a fortnight apart, leading to a marked improvement in mastoid, ear and nose pain, and a fall in CRP within two months to normal levels. She was subsequently commenced on mycophenolate although was not initially able to take this regularly due to side effects. A reduction in oral steroid dose was possible. She has been retreated empirically with rituximab on two occasions with an average of seven months between infusions. She has since felt well enough to return to her university studies. Disease remission has been maintained as demonstrated by a suppression of CRP, which has largely remained below 5 mg/l on subsequent testing.

## 3. Discussion

We feel that this case offers a number of learning points. Firstly, patients presenting with upper respiratory tract symptoms, bilateral middle ear symptoms, and cranial neuropathies which cannot be explained by the effects of infection should have WG high on the list of differential diagnoses. Secondly, this case illustrates response to B-cell depletion in WG with predominant ENT symptoms, cavitating lung lesions and cranial neuropathies where there was no detectable antibody. It is logical to hypothesise that elimination of B cells using rituximab might have a favourable effect on ANCA-associated WG by removing the cells responsible for ANCA production [2–6]. However, the mechanism by which B-cell depletion may act in ANCA-negative cases is less clear. It may be that in these cases, B-cells contribute to disease via additional roles such as antigen presentation or interactions with T cells. Alternatively, high affinity ANCA may be strongly tissue bound resulting in progressive severe disease. Finally, as has been reported previously [7], rituximab was not helpful in preventing the progression of all the disease-specific pathology and subglottic stenosis continued to be a problem in this case. Our patient has improved with operative intervention.

Limited WG, with clinical findings isolated to the upper respiratory tract or the lungs, occurs in approximately one quarter of cases. Although many of these patients (up to 80%) may subsequently develop glomerulonephritis, there are incompletely understood phenotypic differences between subtypes of WG [8]. Patients with limited disease are younger at disease onset and likely to be female. They have longer disease duration on average than patients with systemic disease and a greater likelihood of recurring disease. Patients with limited disease also have a higher prevalence of destructive upper respiratory disease as saddle nose deformity and are less likely to be ANCA positive.

## 4. Conclusion

Our case illustrates that in ANCA-negative WG which relapses at an early stage following conventional cyclophosphamide, there is a role for B-cell depletion despite the lack of a detectable autoantibody target. In such cases, rituximab may be an effective therapeutic option.
